# The interplay of grit, resilience, and burnout among medical students during exams: a cross-sectional study in Mansoura university, Egypt

**DOI:** 10.1038/s41598-026-35125-0

**Published:** 2026-01-29

**Authors:** Yusof Mohamed Omar, Ahmed Abdelmageed, Omar Shaker, Samir Oransa, Mohamed Nasr, Abdelrahman A. Senbel, Mark Messak, Shady Aboheif, Abdel-Hady El-Gilany

**Affiliations:** 1 Faculty of Medicine, Mansoura University, Mansoura, Egypt; 2 Medical Research Group of Egypt, Negida Academy, Arlington, MA, USA; 3 Faculty of Medicine, Delta University, Dakahlia, Egypt; 4 Faculty of Medicine, Helwan University, Helwan, Egypt; 5 Public Health and Community Medicine Department, Faculty of Medicine, Mansoura University, Mansoura, Egypt

**Keywords:** Grit, Resilience, Burnout, Medical students, Egypt, Public health, Quality of life, Psychology

## Abstract

Medical students experience significant burnout during exams, underscoring the need for factors that can buffer its impact. Thus, this cross-sectional study examined the relationship between grit, resilience, and burnout among Mansoura University medical students during the January 2025 exam period. Following a pilot study, an online survey distributed via the faculty’s official student platforms using convenience sampling collected responses from 653 students. Participants completed the Burnout Assessment Tool (BAT-23), Short Grit Scale (Grit-S), and Brief Resilience Scale (BRS). Pearson correlations assessed bivariate relationships, and multivariable linear regression identified independent predictors of burnout. Mean total burnout was 3.62 (range 1–5), and subdomain means were highest for exhaustion (3.96) and cognitive impairment (3.66). Burnout correlated negatively with grit (*r* = − 0.595, *p* < 0.001) and resilience (*r* = − 0.519, *p* < 0.001); grit and resilience were positively correlated (*r* = 0.426, *p* < 0.001). In multivariable regression analysis, both grit (Unstandardized Beta (B) = -0.46, 95% Confidence Interval (CI) [-0.52, -0.39]) and resilience (B = -0.32, 95% CI [-0.38, -0.25]) were independently associated with lower burnout. These findings highlight grit and resilience as significant protective factors against burnout among medical students during exams.

**Supplementary Information:**

The online version contains supplementary material available at 10.1038/s41598-026-35125-0.

## Introduction

Medical education is widely recognized for its rigorous and demanding nature, subjecting students to intense academic pressure. This pressure peaks during exams, often considered the most stressful periods in a medical student’s academic journey^1^. As students navigate the challenges and pressures of medical education, they become particularly vulnerable to experiencing burnout—a pervasive issue plaguing medical students and the healthcare workforce worldwide^2,3^.

As defined by Schaufeli et al.^4^, burnout in students is characterized by exhaustion due to academic demands, a cynical and detached attitude towards their studies, and pervasive feelings of incompetence. Burnout is alarmingly prevalent among medical students, with global estimates indicating that over half experience burnout, and some studies reporting rates exceeding 88%^5^. In Egypt, burnout is particularly prevalent, with studies among medical students consistently reporting high degrees of emotional exhaustion and depersonalization^6,7^. Compounding this issue, Egyptian medical students often neglect self-care and delay seeking mental health support, citing lack of time or a preference for self-reliance, tendencies exacerbated during exams^8,9^.

The demands–resources model suggests that burnout occurs when academic demands exceed students’ available coping resources^10^. During exams, the increased study load and constant pressure due to continuous examinations create an environment where burnout is particularly likely to occur. Alarmingly, burnout during exams has been linked to serious consequences, affecting both cognitive function and ethical decision-making. For one, burnout can impair students’ ability to retain and apply knowledge effectively, potentially leading to worse academic performance^11^. Additionally, studies among U.S. medical students suggest that students experiencing burnout not only perform more poorly on exams, but are also significantly more likely to cheat^12,13^.

The impact of burnout on students extends beyond academic performance, contributing to serious mental health consequences such as depression^14^, substance abuse^15^, and suicidal ideation^16^. Moreover, burnout can often persist into professional life, contributing to higher attrition rates and exacerbating healthcare workforce shortages^17^. In Egypt, where a critical physician shortage persists, burnout among medical students exacerbates the healthcare crisis^18^. Indeed, studies indicate that up to 90% of Egyptian medical students express a desire to emigrate, with burnout being a significant driving factor^3,19^.

Given the high prevalence and significant consequences of burnout, identifying psychological traits that help students manage exam-related stress and mitigate its negative effects is crucial. Two psychological traits, grit and resilience, might fulfill such a role. Grit, defined as passion and perseverance toward long-term goals^20^, can motivate students to persevere through their exams. Meanwhile, resilience, the ability to recover from adversity and adapt to challenges^21^, may enable students to maintain effort throughout exams despite setbacks. Together, these traits may empower students to navigate the pressures of exams, by sustaining motivation and overcoming academic setbacks^22,23^.

Previous research has demonstrated the protective roles of grit and resilience in mitigating burnout across various academic and professional contexts. Grit has been linked to lower burnout rates and better stress management^23^. Similarly, studies among healthcare professionals consistently identify resilience as a key protective factor, with higher resilience predicting lower emotional exhaustion and greater personal accomplishment^24,25^. However, no studies have specifically examined how these factors influence burnout among Egyptian medical students, particularly during exams. Thus, this study aims to fill this gap by exploring the relationship between grit, resilience, and burnout among medical students in Egypt during exams. We hypothesize that students with greater grit and resilience will experience lower levels of burnout, offering insights into how these psychological traits shape well-being and contribute to a more sustainable medical workforce.

## Methods

### Study design and setting

A cross-sectional study with descriptive and correlational components was conducted at the Faculty of Medicine, Mansoura University, during the final exam period in January 2025. The study was approved by the Institutional Research Board (IRB) at the Faculty of Medicine, Mansoura University (R.24.12.2969) and adhered to the Declaration of Helsinki for Medical Research Involving Human Subjects.

###  Pilot study and sample size calculation

A pilot study involving 40 students, with a minimum of 5 students from each academic year, was conducted to assess the reliability of the study’s scales and estimate the minimum required sample size. The standard deviation of the burnout scores was found to be 0.681. Sample size calculations were performed using R programming software with the sampling book package^26,27^. With a 5% margin of error and a 95% confidence level, the required sample size for a finite population of 6,868 individuals was determined to be 646 participants.

### Study population and data collection approach

Data were collected through an online, anonymous, self-administered questionnaire in English, hosted on the FormNX platform. Anonymity was emphasized to encourage candid responses given the personal nature of questions on stress, resilience, and burnout. The survey link was disseminated two weeks into the examination period and remained open until the final exam day. Distribution occurred through the official Telegram channels used by each academic year, ensuring full coverage of the target population. Participation was voluntary, and students were informed of the study objective, anonymity, and the option to view their grit, resilience, and burnout scores after completion.

Inclusion criteria were undergraduate medical students enrolled in the first through fifth years at the Faculty of Medicine, Mansoura University, who accessed and completed the questionnaire during the exam period. Exclusion criteria were interns, postgraduate trainees, and students not enrolled in the Faculty of Medicine of Mansoura University.

### Data collection tool

An anonymous, self-administered online questionnaire was used to collect data on socio-demographics (such as age, sex, academic year, and residence), grit, resilience, and burnout. All sociodemographic questions and included scales were administered in their official English versions, as English is the language of medical instruction in Egypt.

Grit was assessed using the Short Grit Scale (Grit-S), an 8-item valid measure for assessing grit^28^. Example items include “I finish whatever I begin” and “Setbacks don’t discourage me.” The scale is scored on a 5-point Likert scale (1 = not at all like me, 5 = very much like me), the sum of all items is divided by 8 to produce a final score between 1 and 5, with higher scores reflecting greater grit. The scale comprises two subscales—perseverance of effort and consistency of interest—each consisting of four items, with subscale scores following the same range as the total grit score. The Grit-S has displayed an overall excellent internal consistency reliability, with a Cronbach alpha of 0.82 for the total scale, and subscales of consistency of interest (0.84) and perseverance of effort (0.71)^29^.

Resilience was measured using the Brief Resilience Scale (BRS), a 6-item valid measure for assessing resilience^21^. Example items include “I tend to bounce back quickly after hard times” and “It is hard for me to snap back when something bad happens” (reverse-coded). The scale is scored on a 5-point Likert scale (1 = strongly disagree, 5 = strongly agree), with the sum of all items divided by 6 to produce a final score between 1 and 5, with higher scores reflecting higher resilience. The BRS is a reliable instrument displaying Cronbach’s alpha values ranging from 0.80 to 0.91 across four different samples in previous studies^30^.

Burnout was evaluated using the Burnout Assessment Tool Student version (BAT-23), a valid measure for assessing burnout^31,32^. The BAT-23 comprises 23 items across four dimensions: exhaustion (8 items), mental distancing (5 items), cognitive impairment (5 items), and emotional impairment (5 items). Example items include “I feel mentally exhausted by my studies” and “I have trouble concentrating in class.” Responses were rated on a 5-point Likert scale (1 = never, 5 = always). The total score, as well as the scores for each subscale, is calculated by taking the mean of values of all responses, where a higher score reflects a higher level of burnout (range 1–5). The BAT-23 and its subscales show good reliability with Cronbach’s alpha values ranging from 0.90 to 0.92 for the subscales and 0.95 for the total BAT-23^31^. Convergent validity and discriminant validity were demonstrated with other burnout scales, such as the Maslach Burnout Inventory and the Oldenburg Burnout Inventory. The BAT–23 showed the best overall fit, with the most favorable chi-square to degrees of freedom ratio, the highest comparative fit index and Tucker-Lewis index, and the lowest root mean square error of approximation^32^.

Results from the pilot study showed Cronbach’s alpha values of 0.79 for the Grit-S, 0.68 for the BRS, and 0.92 for the BAT, indicating acceptable internal consistency^33^. The slightly lower alpha for the BRS (0.68) was expected given its small number of items, as shorter scales often yield lower reliability estimates. Meanwhile, the BAT’s high alpha (0.92) is consistent with previous studies using the same scale^31^.

### Statistical analysis

Statistical analyses were performed using Jamovi 2.3.28 software^34^. Categorical variables were summarized as frequencies and percentages, while continuous variables were reported as means and standard deviations (SDs). Normality was assessed visually using Quantile-Quantile (Q-Q) plots. Independent Student t-tests and one-way ANOVA were used to compare mean scores of grit, resilience, and burnout across individual and multiple groups, respectively. Pearson’s correlation coefficient was used to examine associations between grit, resilience, and burnout. To examine the relationship between burnout and its predictors, multiple linear regression analysis was conducted. Multicollinearity was assessed using Variance Inflation Factors (VIFs). A p-value of ≤ 0.05 was considered statistically significant.

## Results

### Participant characteristics and differences in grit, resilience, and burnout scores

The study included 653 students with a mean age of 20.3 ± 1.7 years. The cohort was nearly equally distributed by sex (51.3% male, 48.7% female), with a slight majority of students from urban (55.9%) over rural (44.1%) residences. Participants were distributed across all five academic years, with second-year students (32.6%) representing the largest proportion and fourth-year students (8.9%) the smallest (Table 1).

**Table 1:** Study participants’ sociodemographic characteristics and differences in burnout, grit, and resilience (*N* = 653).

Characteristic	*n* (column%)	Burnout mean (SD)^1^	*p*-value	Grit mean (SD)	*p*-value	Resilience mean (SD)	*p*-value
**Sex**							
Male	335 (51.3%)	3.55 (0.67)	**0.009**	2.96 (0.72)	0.59	3.11 (0.7)	**< 0.00**1
Female	318 (48.7%)	3.69 (0.66)		2.93 (0.65)		2.87 (0.65)	
**Age**							
20 and younger	407 (62.3%)	3.58 (0.65)	**0.048**	3 (0.68)	**0.008**	3.02 (0.67)	0.15
21 and older	246 (37.7%)	3.69 (0.69)		2.86 (0.68)		2.94 (0.7)	
**Academic year**							
1 st year	88 (13.5%)	3.46 (0.63)	0.102	3.01 (0.57)	0.524	3.08 (0.66)	0.262
2nd year	213 (32.6%)	3.63 (0.67)		2.98 (0.7)		3.03 (0.69)	
3rd year	160 (24.5%)	3.62 (0.68)		2.95 (0.72)		2.96 (0.69)	
4th year	58 (8.9%)	3.61 (0.67)		2.93 (0.69)		3.02 (0.65)	
5th year	134 (20.5%)	3.71 (0.69)		2.87 (0.69)		2.9 (0.69)	
**Residence**							
Rural	288 (44.1%)	3.64 (0.67)	0.418	2.94 (0.67)	0.868	2.97 (0.68)	0.413
Urban	365 (55.9%)	3.6 (0.67)		2.95 (0.7)		3.01 (0.68)	

The overall mean scores for burnout, grit, and resilience were 3.62 (SD = 0.67), 2.95 (SD = 0.68), and 2.99 (SD = 0.68), respectively Among core burnout symptoms, exhaustion had the highest mean score (3.96 ± 0.74), while mental distancing had the lowest (3.21 ± 0.79) (Table 2).

**Table 2:** Mean scores for primary study variables.

Variable	Mean (SD)
Burnout (overall)	3.62 (0.67)
Exhaustion	3.96 (0.74)
Mental distancing	3.21 (0.79)
Cognitive impairment	3.66 (0.92)
Emotional impairment	3.45 (0.93)
Grit (overall)	2.95 (0.68)
Perseverance of effort	3.31 (0.78)
Consistency of interest	3.42 (0.87)
Resilience (overall)	2.99 (0.68)

Females reported significantly lower resilience scores (2.87 vs. 3.11, *p* < 0.001) as well as significantly higher burnout scores (3.69 vs. 3.55, *p* = 0.009) compared to males. Similarly, older students reported significantly lower grit scores (2.86 vs. 3.00, *p* = 0.008) and, in turn, significantly higher burnout scores (3.69 vs. 3.58, *p* = 0.048) than younger students (Table 1).

### Correlation between grit, resilience, and burnout

Burnout was negatively correlated with both grit (*r* = −0.595) and resilience (*r* = −0.519), indicating that students with higher levels of grit and resilience experienced lower burnout. In terms of grit, consistency of interest correlated more negatively with burnout than persistence of effort (*r* = −0.576 vs. −0.406). Additionally, grit and resilience were positively correlated (*r* = 0.426), suggesting that students with greater grit also tended to have higher resilience (Table 3). Among the core burnout symptoms, grit showed the strongest negative correlation with cognitive impairment (*r* = −0.6), followed by exhaustion (*r* = −0.513). Resilience was most strongly correlated with emotional impairment (*r* = −0.507), followed by exhaustion (*r* = −0.439).

**Table 3:** Correlation between burnout, grit, and resilience scores among study participants. All correlations marked with * are statistically significant (*p* < 0.001).

	Total burnout	Exhaustion	Mental distancing	Cognitive impairment	Emotional impairment	Resilience
Grit	−0.595*	−0.513*	−0.438*	−0.6*	−0.354*	0.426*
Perseverance	−0.406*	−0.314*	−0.342*	−0.452*	−0.211*	0.370*
Consistency	−0.576*	−0.530*	−0.386*	−0.544*	−0.37*	0.342*
Resilience	−0.519*	−0.439*	−0.311*	−0.395*	−0.507*	—

### Predictors of burnout

In the linear regression analysis, the VIFs for all predictors ranged from 1 to 2, indicating no significant multicollinearity. The model explained 44.6% of the variance in burnout (R² = 0.446, Adjusted R² = 0.439). Grit (B = −0.46, 95% CI [−0.52, −0.39], *p* < 0.001) and resilience (B = −0.32, 95% CI [−0.38, −0.25], *p* < 0.001) were identified as significant predictors of burnout, with higher levels of both traits associated with lower burnout scores (Table 4).

**Table 4:** Linear regression analysis of factors associated with burnout among the medical students of Mansoura university during exams. Significant values are in bold.

Variables	Unstandardized beta (B) [95% CI]	Standardized beta (β) [95% CI]	*p*-value
Age (continuous)	0.00 [−0.04, 0.04]	0.01 [−0.09, 0.10]	0.866
Sex (Male = 0, Female = 1)	0.05 [−0.03, 0.13]	0.04 [−0.02, 0.09]	0.249
Academic year (Ordinal)	0.01 [−0.04, 0.06]	0.02 [−0.08, 0.12]	0.683
Residence (Urban = 0, Rural = 1)	0.02 [−0.06, 0.10]	0.02 [−0.04, 0.07]	0.588
Grit	−0.45 [−0.51, −0.39]	−0.46 [−0.52, −0.39]	**< 0.001**
Resilience	−0.31 [−0.37, −0.25]	−0.32 [−0.38, −0.25]	**< 0.001**

## Discussion

The present study explored the relationship between grit, resilience, and burnout in medical students during exams. We found that both grit and resilience were significant protective factors, demonstrating a strong negative correlation with burnout. Furthermore, we found that female students reported significantly lower resilience, which was associated with higher burnout, while older students exhibited lower grit, also correlating with increased burnout. These findings highlight the critical role of these psychological traits in mitigating exam-related stress and provide a clear rationale for targeted interventions.

The students in our study reported significant levels of burnout, with exhaustion and cognitive impairment being the most prevalent symptoms. These findings align with a study among medical students in Pakistan^35^, which also identified exhaustion as the most frequently reported symptom, followed by cognitive impairment. Similarly, previous studies among medical students in Egypt have shown that those experiencing a heavy academic burden reported significantly higher levels of burnout, highlighting the impact of academic pressure on student well-being^6,36^.

In this study, female students exhibited lower resilience, which was linked to higher burnout, while older students demonstrated lower grit, correlating with increased burnout. These findings align with previous research on Egyptian medical students^30,37^, where female students consistently reported lower resilience, contributing to greater stress and emotional impairment. This is also consistent with findings among healthcare professionals during the COVID-19 pandemic, where female gender was a significant predictor of higher emotional exhaustion and was also correlated with lower resilience^25^. However, our results contrast with those of a study on Korean medical students^38^, where older students showed lower burnout and greater grit, though the difference compared to younger students was not statistically significant.

The lower resilience observed in female students may be partly explained by intrinsic psychological factors, as research on Egyptian university students indicates that females often score higher on neuroticism, a trait linked to lower resilience^39^. This potential vulnerability may be further exacerbated by career-specific sociocultural stressors, as female medical students and physicians in Egypt must navigate a traditionally male-dominated clinical and academic hierarchy^40^, while also contending with cultural expectations to prioritize marriage and domestic responsibilities^41^. The cumulative burden of these gender-based stressors likely contributes to greater stress, making female students more vulnerable to burnout under the high-stakes pressure of exams^9,30^. Meanwhile, older students’ lower grit during exams may stem from competing responsibilities, such as family and financial obligations, which can reduce their ability to persist through academic challenges. A study on medical students in the United States similarly found that older students reported lower grit and, consequently, less tolerance for uncertainty^42^.

These findings underscore the need for targeted interventions. Female students could benefit from multi-session resilience-building programs, which have been shown to enhance resilience, grit, and quality of life among Egyptian nursing students^43^. These workshops could incorporate evidence-based educational components which systematic reviews have found to be significantly effective such as cognitive re-framing, goal-setting, deep breathing, and mindfulness meditation^44^. Meanwhile, tailored support systems and mentorship programs for older students, particularly during exam periods, could help mitigate the impact of external responsibilities on their academic performance and mental wellbeing.

In our study, burnout was significantly negatively correlated with both grit and resilience, suggesting that students with higher levels of these traits experienced lower levels of burnout. This aligns with previous longitudinal studies among medical students, which also found a negative relationship between burnout and grit^22,45^. Moreover, grit has been linked to improved academic performance, underscoring its role in managing stress and maintaining focus during challenging periods^46^. Similarly, a study among pharmacy students reported lower stress levels during exams among more resilient students^47^. Additionally, studies have demonstrated that this protective effect extends to broader mental health outcomes, with resilience being negatively associated with depression, stress, and anxiety^48,49^, further highlighting its protective role in alleviating the psychological strain often experienced during high-stress situations like exams.

The significant negative correlation between burnout and grit/resilience during exams suggests that students with higher levels of these traits are better equipped to cope with the intense pressures of high-stakes assessments. Grit helps students maintain motivation and persistence despite setbacks, while resilience enables them to adapt to stress and recover more effectively from adversity^20,50^. Together, these traits likely act as protective factors, reducing emotional exhaustion and preventing feelings of overwhelm. Conversely, students with lower levels of grit and resilience may struggle to meet the demands of exams, leaving them more susceptible to burnout.

This notion is further supported by the positive correlation between resilience and grit observed in our study. Previous research suggests that grit, being a dynamic trait, can be cultivated to enhance resilience and reduce academic stress during challenging periods^51^. While some studies have not found a clear connection between grit and resilience^52^, the broader body of evidence strongly supports their interdependence, especially in the context of managing stress and overcoming adversity^53,54^. These findings underscore the importance of fostering both grit and resilience in students to help them navigate the challenges of medical education and reduce the risk of burnout.

In light of the significant challenges facing Egypt’s healthcare system, where many burnt out residents and medical students report plans to emigrate upon graduation^3,19^, this study highlights the potential of cultivating resilience and grit as effective strategies to combat burnout and improve academic performance. By fostering these traits, students can develop stronger tools for managing stress and maintaining their well-being, particularly during high-pressure situations like exams. We recommend that medical faculties and policymakers consider the formal integration of resilience- and grit-building modules within the core medical curriculum, rather than as optional, ad-hoc support. A recent systematic review by Calo et al. found a significant overlap in the components of effective programs, suggesting that curricular modules could be designed to develop interconnected skills such as perseverance, time management, and goal identification, alongside strategies for addressing fear of failure and re-framing negative thoughts^44^. While providing immediate support to medical students during exams may be challenging, a curricular-based policy of fostering these traits early on offers a sustainable, preventative approach to mental health. This would not only help students manage stress but also empower them to excel academically and professionally, ultimately contributing to a healthier, more resilient healthcare workforce in Egypt by directly addressing the factors that drive physician attrition.

## Strengths and limitations

This study has several strengths, including the validated and reliable tools use to measure burnout, grit, and resilience. Furthermore, the study assesses these traits during exams, a period often understudied due to logistical constraints. Additionally, allowing students to view their burnout scores at the end of the survey may have contributed to raising mental health awareness among participants.

However, several limitations should be considered. The cross-sectional design limits causal inference between grit, resilience, and burnout. Reliance on self-reported data may have introduced social desirability bias or inaccurate self-assessment. Although the survey was distributed through the faculty’s official Telegram groups, not all students may have been actively using these platforms during the exam period, which may have contributed to sampling bias particularly if students experiencing higher burnout were less likely to participate. The use of English-only instruments, while appropriate given that English is the language of medical instruction, may still have posed challenges for some students and could have influenced response accuracy. Additionally, some potentially relevant demographic or clinical variables such as diagnosed mental illness, acute illness during exams, or prior mental health treatment were not collected and may have introduced residual confounding. As the study was conducted in a single institution, the findings may not be generalizable to all medical students in Egypt. Finally, because data were collected during examinations, the results may not reflect stress or coping patterns during less demanding periods. Future research should adopt longitudinal, multi-center approaches and include a broader set of demographic and clinical variables to improve generalizability.

## Conclusion

This study found that grit and resilience are significant protective factors associated with lower burnout among medical students during exams. These findings provide an evidence-based rationale for medical educators and policymakers to support and consider the formal integration of interventions that foster these psychological traits within the medical curriculum. Such a strategic approach offers a sustainable, preventative model to enhance student well-being and, ultimately, may help address the challenge of burnout-driven workforce attrition. However, further research using longitudinal designs and more inclusive sampling methods is needed to better understand the causal relationships between these factors.

## Supplementary Information

Below is the link to the electronic supplementary material.

Supplementary Material 1

## Data Availability

The dataset supporting the findings of this study has been attached as a supplementary file with this submission.

## Abbreviations

Grit-S: Short grit scale
BRS: Brief resilience scale
BAT: Burnout assessment tool
SD: Standard deviation
STROBE: Strengthening the reporting of observational studies in epidemiology
VIFs: Variance inflation factors
